# Highly Transparent, Mechanically Robust, and Conductive Eutectogel Based on Oligoethylene Glycol and Deep Eutectic Solvent for Reliable Human Motions Sensing

**DOI:** 10.3390/polym16192761

**Published:** 2024-09-30

**Authors:** Zhenkai Huang, Jiahuan Xie, Tonggen Li, Liguo Xu, Peijiang Liu, Jianping Peng

**Affiliations:** 1 School of Materials and Energy, Foshan University, Foshan 528000, China; hzk@fosu.edu.cn (Z.H.);; 2 School of Environmental and Chemical Engineering, Foshan University, Foshan 528000, China; 3 Reliability Physics and Application Technology of Electronic Component Key Laboratory, The 5th Electronics Research Institute of the Ministry of Industry and Information Technology, Guangzhou 510610, China; 4 College of Light Chemical Industry and Materials Engineering, Shunde Polytechnic, Foshan 528333, China; 21099@sdpt.edu.cn

**Keywords:** eutectogel, deep eutectic solvent, strain sensor, wearable device, human motions sensing

## Abstract

Recently, eutectogels have emerged as ideal candidates for flexible wearable strain sensors. However, the development of eutectogels with robust mechanical strength, high stretchability, excellent transparency, and desirable conductivity remains a challenge. Herein, a covalently cross-linked eutectogel was prepared by exploiting the high solubility of oligoethylene glycol in a polymerizable deep eutectic solvent (DES) form of acrylic acid (AA) and choline chloride (ChCl). The resulting eutectogel exhibited high transparency (90%), robust mechanical strength (up to 1.5 MPa), high stretchability (up to 962%), and desirable ionic conductivity (up to 1.22 mS cm^−1^). The resistive strain sensor fabricated from the eutectogel exhibits desirable linear sensitivity (GF: 1.66), wide response range (1–200%), and reliable stability (over 1000 cycles), enabling accurate monitoring of human motions (fingers, wrists, and footsteps). We believe that our DES-based eutectogel has great potential for applications in wearable strain sensors with high sensitivity and reliability.

## 1. Introduction

Flexible and stretchable ionic conductors, including gels and elastomers, have recently gained significant attention for their applications in wearable electronic devices, such as soft sensors [1,2,3], flexible transducers [4,5,6], and flexible luminescent devices [7,8,9,10]. Conductive hydrogels have been widely utilized in wearable electronic devices [11,12]. However, hydrogels encounter significant challenges, including unavoidable water evaporation and freezing at sub-zero temperatures [13]. Such dehydration and freezing adversely affect the mechanical properties and conductivity of hydrogels, impeding their suitability for wearable electronic applications.

To address these issues, various anti-freezing strategies have been proposed. These include lowering the freezing point by incorporating salts [14,15] or organic solvents [16,17] and integrating hydrophobic and hydrophilic structures to inhibit ice recrystallization and growth [18]. Recently, the development of ionic liquid gels has been introduced as a novel approach to mitigate water evaporation by incorporating ionic liquids into the polymer network [19,20]. Ionic liquids, as non-volatile salts, possess advantageous properties but are hampered by their high cost and complex synthesis and processing requirements [21,22,23].

Recently, eutectogels composed of deep eutectic solvents (DESs) have emerged as a novel type of ionic gel, offering advantages such as superior conductivity, low volatility, thermal stability, non-toxicity, and cost-effectiveness, thereby surpassing hydrogels and ionogels [24,25]. DESs are synthesized through a straightforward, cost-effective process involving the heating and mixing of a hydrogen bond acceptor (HBA) and a hydrogen bond donor (HBD) without the need for purification [24]. The strong interactions, such as hydrogen bonding and van der Waals forces, between the components result in a melting point significantly lower than that of the individual components [26,27]. The low freezing point and low volatility of DESs endow eutectogels with exceptional anti-freezing and anti-drying characteristics, making eutectogel an ideal alternative for conductive hydrogel. Moreover, DES-based eutectogels demonstrate enhanced mechanical properties and conductivity, making them suitable for applications in strain and pressure sensing [28].

Polymerizable DESs can be cured into eutectogels through in situ polymerization of the double bonds present in the DES monomer. For instance, He et al. synthesized a polymerizable DES using acrylic acid and choline chloride as the hydrogen bond donor and acceptor, respectively [29]. Similarly, Lian et al. produced a eutectogel by dissolving a polymerizable monomer in a conductive DES and polymerizing the monomer [30]. These methods are among the most commonly reported for obtaining conductive eutectogels. Additionally, one-pot polymerization allows for the fabrication of gels in various shapes by injecting gel precursor solutions into different molds [31]. Hu’s group developed a stepwise reinforcement strategy to create eutectogels with enhanced mechanical properties [32]. This method continuously modulates and optimizes the poly(vinyl alcohol) network through an initial annealing step followed by solvent exchange with the DES, resulting in a significant strengthening of the polymer network through rigid crystal domain cross-linking. However, the preparation process is complex and limits the ability to easily shape the gel in terms of size and form. In contrast, Wu’s group reported a hydrophobic conductive eutectogel created via one-step photoinitiated copolymerization in a hydrophobic deep eutectic solvent [33]. This eutectogel demonstrates excellent transparency, stretchability, low hysteresis, and adjustable adhesion, making it suitable for air/underwater mechanosensing. However, its mechanical properties are inadequate to withstand severe deformation loads in practical applications. The current challenge remains to develop a one-pot polymerization of eutectogel that achieves both high ionic conductivity and robust mechanical properties.

Herein, P(AA-ChCl)/oligoethylene glycol eutectogels were prepared by leveraging the high miscibility of the P(AA-ChCl) matrix and oligoethylene glycol, which ensured the homogeneous formation of the eutectogels and thus exhibited remarkable optical transparency. The covalently cross-linked polymer DES network structure and hydrogen-bonding energy-dissipation mechanism contribute to the high mechanical properties of the obtained eutectogels, which exhibit tensile strength up to 1.5 MPa and toughness up to 2.79 MJ m^−3^, along with notable stretchability, as evidenced by elongation at break of up to 962%. With our design, we achieved robust mechanical properties using a simple one-pot method. Moreover, the mobile ChCl molecules surrounding the polymer network endow the eutectogel with excellent ionic conductivity (up to 1.22 mS cm^−1^). The conductivity and elasticity of the eutectogel render it suitable for use as a flexible strain sensor, exhibiting accurate sensing properties for human motion information (e.g., finger, wrist, and knee movements). The findings of this study offer a promising avenue for the development of eutectogels with enhanced stretchability, transparency, and adhesive properties, which could be utilized in flexible wearable sensor devices.

## 2. Results and Discussion

### 2.1. Design and Preparation of the P(AA-ChCl)/OEG Eutectogels

The eutectogels were designed based on the principle of mixing a polymerizable deep eutectic solvent (DES) with oligoethylene glycol (OEG), followed by photoinitiated in situ polymerization and cross-linking to form a robust polymer network. The polymerizable acrylic acid (AA)-choline chloride (ChCl) DES was chosen as the monomer for the polymer network of eutectogels. It has been well documented that acrylates (including acrylic acid, methacrylic acid, acrylamide, and itaconic acid) can act as hydrogen bond acceptors (HBAs) and ChCl can act as a hydrogen bond donor (HBD) [29,34]. When the hydrogen bond acceptor (HBA) and hydrogen bond donor (HBD) were mixed in appropriate molar ratios, a homogeneous and transparent polymerizable DES was formed [29,34]. In this work, the AA-ChCl DES was synthesized by screening several molar ratios of AA and ChCl. OEG contains a substantial number of hydroxyl groups (-OH), which can facilitate numerous hydrogen bonding interactions with carboxyl groups (-COOH) present on the polymer matrix of eutectogel. This enhances the compatibility of OEG with the polymer matrix and improves the stability of eutectogels. Moreover, the exceptional stability and non-volatility of OEG endow eutectogels with remarkable non-volatility and non-leakage features. A widely used diacrylate cross-linker, ethylene glycol dimethacrylate (EGDMA), was chosen to serve as the covalent cross-linker in the polymer matrix of eutectogels [35,36]. The density of chemical cross-linking sites in the polymer network of the eutectogel can be significantly affected by modulating the amount of cross-linking agent. Consequently, the tensile properties and mechanical strength of the eutectogel can be modified.

**Figure 1:**
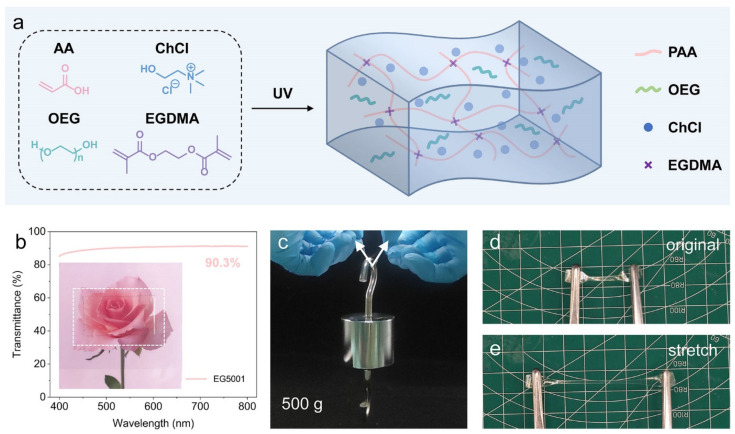
Schematic illustration for the design of the P(AA-ChCl)/OEG eutectogel. (**a**) The preparation of the P(AA-ChCl)/OEG eutectogel. A DES was first synthesized by mixing AA and ChCl with a fixed molar ratio. The hydrogen bonding interactions and coulombic interactions between P(AA-ChCl) polymer matrix and OEG contributed to the formation of a covalently cross-linked eutectogel. (**b**) Transmittance spectrum of the eutectogel with a film thickness of 1.7 mm. An average transmittance of over 90% was recorded in the visible range (400–800 nm). Inset: photograph of the film over an image of flowers. (**c**) Photograph of a eutectogel (20 mm × 5 mm × 1.8 mm) holding up a weight of 0.5 kg. (**d**,**e**) Photographs of a dumbbell-shaped eutectogel (20 mm × 2 mm × 1.8 mm) before and after being stretched to four times its original length. The side length of the background grid is 10 mm.

The synthesis methodology for P(AA-ChCl)/OEG eutectogels is illustrated in Figure 1a. It is well documented that AA, serving as an HBD, and ChCl, acting as an HBA, form stable, transparent, and deep eutectic solvents at a molar ratio of 2:1 [29,34]. Consequently, this study maintained a fixed molar ratio of 2:1 for AA to ChCl. For simplicity, the eutectogels with varying proportions are denoted as EGxxyy, where “xx” denotes the mass fraction of OEG in the dispersive medium and “yy” specifies the millesimal molar ratio of EGDMA. For instance, EG5001 signifies an OEG mass fraction of 50% and an EGDMA molar ratio of 0.1%. A comprehensive overview of the precise proportions of all the eutectogels employed in this study is provided in Table S1. To prepare the precursor solution, a predetermined ratio of AA to ChCl 2:1 was mixed to form a DES solution. Subsequently, the OEG was dissolved into the DES, yielding a homogeneous, transparent, and slightly viscous precursor solution. Subsequently, the precursor solution was cast into a laboratory-made mold and sealed with a pet film. The precursor solution was polymerized in the molds to form P(AA-ChCl)/OEG eutectogels via a photoinitiated free radical polymerization process. As shown in Figure 1b, the P(AA-ChCl)/OEG eutectogels with varying ratios exhibit substantial transparency, with a transmittance exceeding 90%. The high transmittance of the eutectogels can be attributed to the excellent miscibility of OEG with P(AA-ChCl). The hydrogen bonding interactions, coulombic interactions, and covalent cross-linking of the polymer network are also illustrated in Figure 1a, which contribute to the toughness, elasticity, and adhesive properties of the eutectogels. The as-prepared eutectogel samples exhibited robust mechanical strength, as evidenced by their ability to support a weight of 0.5 kg (Figure 1c) and undergo tensile deformation (Figure 1d).

### 2.2. Characteristic and Anti-Freezing Properties of the P(AA-ChCl)/OEG Eutectogels

**Figure 2:**
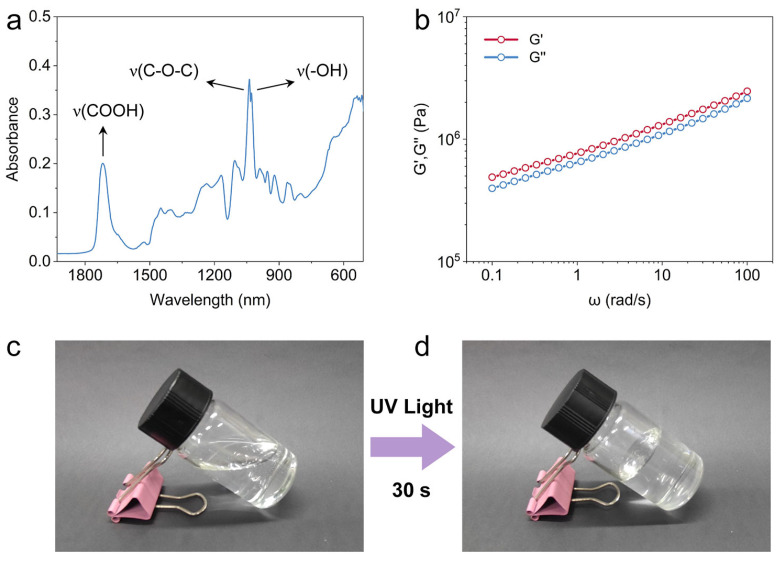
Characteristic of the P(AA-ChCl)/OEG eutectogels. (**a**) ATR-FTIR spectra of the P(AA-ChCl)/OEG eutectogel with the proportion of EG5001. (**b**) Variations of storage modulus G′ and loss modulus G″ of the eutectogel as a function of angular frequency at 25 °C. The precursor solution gels to an immobile eutectogel before (**c**) and after only 30 s of photoinitiated polymerization (**d**).

Fourier transform infrared spectroscopy (FT-IR) confirms the successful preparation of covalently cross-linked eutectogels. As shown in Figure 2a and Figure S1, the removal of the C=C signal at 1635 cm^−1^ in the FTIR spectrum of the eutectogel indicated that the AA-ChCl DES monomer was successfully polymerized [37]. In addition, the distinctive absorption bands appearing at 1720 cm^−1^ and 1038 cm^−1^ in the FTIR spectrum can be attributed to the carboxylic acid groups capable of acting as hydrogen-bonding donors and the hydroxy groups of the OEG. The gelation of the eutectogel was mainly due to hydrogen bonding and coulombic interactions between AA/ChCl and ChCl/OEG. Meanwhile, the formation of the eutectogel was also confirmed by the rheological characteristic (Figure 2b). The rheological results showed that the eutectogel exhibited gel properties with a storage modulus (G′) larger than the loss modulus (G″) at different frequencies. It is anticipated that the G’ value of the eutectogel can reach 10^6^ Pa. In this eutectogel, synergistic interactions are developed between the carbonyl group of acrylic acid and the choline chloride ion, as well as the hydroxy group of OEG and the choline chloride ion. The excellent dissolution properties of OEG in the DES are attributed to the formation of hydrogen bonds between the anions of the DES and the hydroxyl group of OEG. Furthermore, the coulombic interactions between charged moieties contributed to the excellent mechanical properties of the eutectogels [24]. The P(AA-ChCl) network cross-linking by EGDMA can provide a robust reversible energy dissipation network for toughening the eutectogel. It can, therefore, be concluded that a tough eutectogel can be successfully prepared by interpenetrating OEG into the P(AA-ChCl) network (Figure 1a).

Notably, the curing process of the photoinitiated polymerization of the eutectogel is rapid, with the prepared homogeneous eutectogel precursor solution undergoing curing within 30 s and exhibiting a solidified state that precludes its ability to flow like a liquid (Figure 2c,d). The rapid gelation process is advantageous in accelerating the synthesis and production of gels, while also offering a promising avenue for light-curing 3D printing [38,39,40]. Moreover, OEG is a common anti-freeze with a very low freezing point, so mixing OEG into the eutectogel will prevent it from freezing at low temperatures. Thus, the P(AA-ChCl)/OEG eutectogel can remain soft and flexible at −10 °C and can be bent at will (Figure S2). The anti-freezing feature of the eutectogel indicates that the eutectogel exhibits considerable low-temperature stability, which is much better than the corresponding conventional hydrogel at low temperatures.

### 2.3. Mechanical Properties of the P(AA-ChCl)/OEG Eutectogels

**Figure 3:**
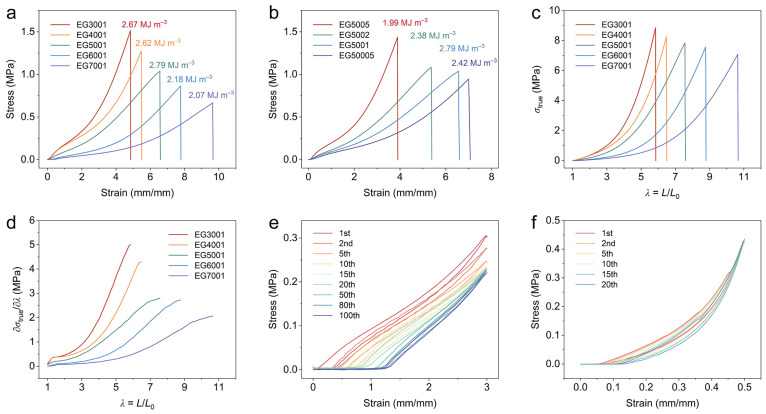
Mechanical properties of P(AA-ChCl)/OEG eutectogels. (**a**) Stress–strain curves with varied OEG mass fractions. (**b**) Stress–strain curves of eutectogels prepared with different molar ratios of EGDMA. (**c**) True stress and (**d**) differential modulus changes with elongation. (**e**) Cyclic tensile release at 300% strain for durability. (**f**) Cyclic compression–recovery at 50% strain for resilience.

By tuning the molar fraction of OEG and EGDMA, a series of eutectogels with different mechanical properties was investigated. The eutectogels with different proportions exhibited excellent mechanical strength (from 0.66 to 1.52 MPa) and ductility (elongation at break up to 962%), as shown in Figure 3a. As OEG mass fraction decreased from 70% to 30%, tensile stress ranged from 0.66 MPa to 1.52 MPa, while elongation varied from 962% to 484% (Table S2). As expected, decreasing OEG content boosts maximum tensile stress and Young’s modulus and reduces elongation due to the reduced plasticizing effect on the P(AA-ChCl) matrix. Moreover, increasing EGDMA content would lead to denser cross-linking, resulting in higher Young’s modulus and lower elongation of the eutectogel. Similarly, lower cross-linker content resulted in a decrease in the mechanical strength and an increase in the stretchability of the eutectogel (Figure 3b and Table S2). EG5001 exhibited the highest toughness (2.79 MJ m^−3^), ultimate stress (1.04 MPa), and elongation (655%). Hydrogen bonding and Coulomb interactions enhance eutectogel stability, preventing leakage under strain. All eutectogels displayed distinct strain-stiffening, evident in true stress-elongation and differential modulus–elongation curves (Figure 3c,d, Figures S3 and S4) [41,42]. Notably, the differential modulus–elongation curves of the eutectogels reveal a unique sigmoid shape that closely mirrors the deformation response observed in natural skin materials [2,11]. Among them, the EG5001 sample showed a 15-fold increase in modulus during tensile testing.

To further explore the viscoelastic properties of the eutectogel, stretch-releasing tests with 300% tensile strain were conducted, revealing residual strain and hysteresis ratio (Figure 3e). Successive cyclic tensile tests at this strain showed a gradual decline in tensile strength with increased cycles, indicating that the partially broken noncovalent interactions in the network were unable to recover promptly. The loading–unloading curves under set strains exhibited hysteresis loops and residual strains. As the number of cycles increased, both the hysteresis ratio and maximum strength in these cycles decreased (Figure S5). After 80 cycles, the residual strain stabilized around 40%, and hysteresis in tensile strength was no longer noticeable. Tensile test hysteresis tied to disruption of reversible noncovalent interactions (e.g., hydrogen bonding, Coulomb) prevalent in noncovalent gel systems [43,44]. Notably, EG5001 also exhibits good resilience and fatigue resistance in the continuous compression mode. Under a compressive strain of 50%, the compress–release curves recorded during different cycles almost overlapped each other (Figure 3f, Figures S6 and S7). The good resilience and cyclic stability of EG5001 could be attributed to the cross-linked network and the multiple reversible interactions formed between the copolymer P(AA-ChCl) polymer matrix and OEG [45,46,47].

### 2.4. Electrical Sensing Performance

**Figure 4:**
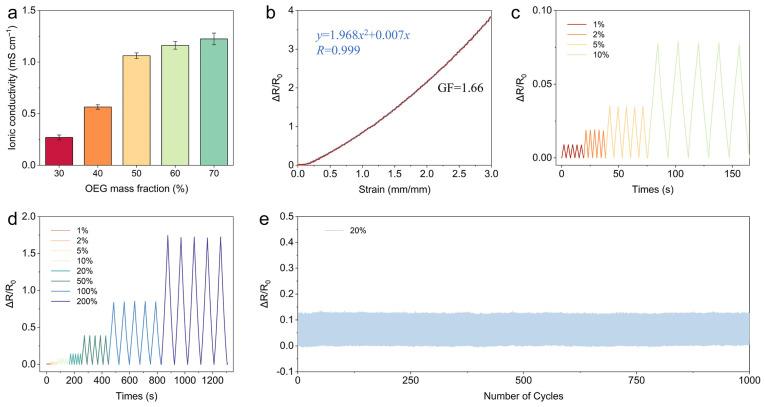
Electrical sensing performance of the P(AA-ChCl)/OEG eutectogels. (**a**) Ionic conductivities of the eutectogels with different OEG mass fractions. (**b**) EG5001 strain sensor’s resistance changes vs. tensile strain. Real-time monitoring at strains (**c**) 1–5% and (**d**) 20–200%. (**e**) Electrical resistance signals under 1000 cycles of 20% strain.

Besides mechanical properties, ionic conductivity is crucial for eutectogel sensors. Conductivity stems from free DES (ChCl) ions in P(AA-ChCl)/OEG eutectogels. EIS measurements were used to study the ionic conductivity of eutectogels with different OEG contents (Figure S8). Ionic conductivity is calculated as σ = L/RS, with L = sample thickness, R = bulk resistance from Nyquist plot, and S = sample cross-section. As shown in Figure 4a, the ionic conductivity of this eutectogel exhibits a notable increase from 0.27 to 1.22 mS cm^−1^ as the OEG mass fraction rises from 30 to 70%. This phenomenon can be attributed to the enhanced mobility of the conducting ion. The EG5001 eutectogel, which exhibits excellent mechanical properties, displays a moderate ionic conductivity of 1.06 mS cm^−1^, indicative of exemplary comprehensive performance. The decomposition voltage of EG5001 obtained from the linear sweep voltammetry curve exceeded 3.6 V (Figure S9), demonstrating a wide electrochemical window and high electric stability of the eutectogel. This makes it an optimal candidate for utilization as a flexible strain sensor.

By monitoring the real-time resistance of the eutectogel under tensile strain, a resistive flexible strain sensor capable of measuring the tensile strain can be obtained. In order to illustrate the potential of our eutectogel as flexible sensors, we constructed flexible strain sensors based on EG5001 by connecting both ends of the eutectogel with wires to an electrochemical workstation. The size of the EG5001 eutectogel was 40 mm × 10 mm × 1.7 mm. As shown in Figure 4b, real-time resistance change (Δ*R*/*R*_0_) is monitored at 0–300% strain, with initial resistance ~1.2 × 10^5^ ohms. The gauge factor (GF) is 1.66 within 200–300% strain. The sensor generates reversible, repeatable signals at low (1–10%) and high (20–200%) (Figure 4c,d) strains, including 1% ultralow strain. The 1000 cycles at 20% strain show stable (Figure 4e), reproducible Δ*R*/*R*_0_ signals with negligible decline, indicating long-term durability. Excellent sensing performance is due to the eutectogel’s mechanical properties and reliable ionic conductivity. When the eutectogel is stretched, its cross-sectional area decreases, resulting in increased resistance. Simultaneously, the length of the eutectogel increases, further contributing to the rise in resistance. The interplay of these two factors causes a significant increase in the eutectogel’s resistance under tensile deformation, enhancing the sensor’s sensitivity. Consequently, this eutectogel-based resistive strain sensor can be considered a promising candidate for use in flexible electronics.

### 2.5. Strain Sensor Application

**Figure 5:**
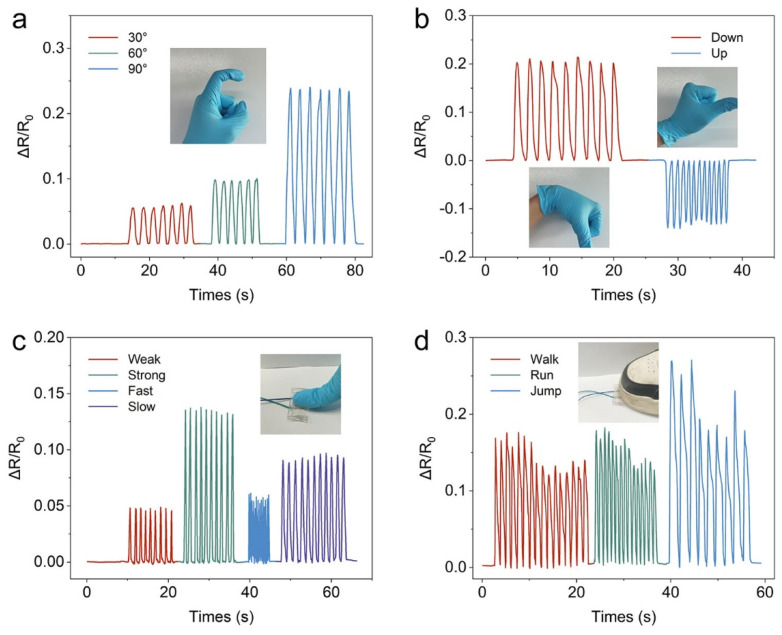
Human motion detection of the eutectogel-based resistive strain sensor: (**a**) Relative electrical resistance signals during finger bending at different angles. (**b**) Relative resistance changes of the eutectogel-based strain sensor during wrist bending up and down. (**c**) Signals of the resistive pressure sensor when the frequency and pressure are different. (**d**) Variation of the resistance response of resistive pressure sensors under different motion states of the human body (e.g., walking, running, and jumping).

To verify the feasibility of our eutectogel-based resistive strain sensor in flexible electronic devices, a series of human motion detection applications are demonstrated in Figure 5. The sensors were attached to different joints to analyze human motion. As a result, the signals of the motion at different angles are recorded with an obvious characteristic (Figure 5a). Relative resistance rises with finger bending, returning to original when straightened. Notably, the relative resistance remains constant when the finger is straightened. Similar motion detections were applied to the wrist, and a clear action response was also obtained (Figure 5b). Different sensor deformations result in varying relative resistive changes. When the sensor is attached above the wrist, bending the wrist downward causes the sensor to stretch, increasing its length and resistance. Conversely, bending the wrist upward compresses the sensor, reducing its length and resistance. As a result, the response curve in Figure 5b appears to increase and then decrease in resistance. The sensor detects pressure types via VHB on the index finger beyond large joint movements. Different intensities and rates of pressure (weak, strong, fast, slow) corresponded to different shapes of the signal (Figure 5c). When pressure sensors are placed underneath shoes, they can be used to gather information about a volunteer’s movements as they walk. In particular, the signals collected by the sensors can be clearly distinguished when a volunteer is walking, running, and jumping (Figure 5d). In Figure 5c,d, the sensors are subjected to lateral compression, which is different from the axial tensile strain shown in Figure 5b. At this point, the axial dimensions of the sensor remain relatively unchanged while the cross-sectional area decreases, resulting in a significant increase in resistance. Thus, the sensors in Figure 5c,d exhibit increased resistance under compression. These encouraging results show that this P(AA-ChCl)/OEG eutectogel can be a desirable wearable sensor for human motion sensing applications.

## 3. Conclusions

In conclusion, we have successfully developed a transparent and mechanically robust P(AA-ChCl)/OEG eutectogel using a straightforward one-pot photoinitiated polymerization process. The resulting eutectogels display an array of impressive properties, including transparency, exceptional mechanical strength, anti-freeze capabilities, and conductivity, all attributed to the multiple reversible interactions and covalent cross-linking within the polymer matrix. Additionally, a wearable resistive flexible strain sensor based on these eutectogels was assembled, demonstrating accurate responses to a broad range of human motions. This study introduces a novel method for creating stretchable and transparent eutectogels. Considering the remarkable and multifunctional attributes, we anticipate that our eutectogels will find promising applications in wearable electronics, soft robotics, and human–machine interfaces.

## Data Availability

The data supporting this work are available from the corresponding authors upon reasonable request.

## Supplementary Materials

The following supporting information can be downloaded at: https://www.mdpi.com/article/10.3390/polym16192761/s1, Figure S1: FT-IR spectra of the P(AA-ChCl)/OEG eutectogel with the proportion of EG5001 from wavelength 500 to 4000 cm^−1^; Figure S2: At temperatures of −10 °C, the eutectogel remains its flexibility and bends naturally under gravity; Figure S3: Corresponding true stress-strain curves of the eutectogels with different crosslinker fraction as a function of elongation; Figure S4: Corresponding differential modulus-strain curves of the eutectogels with different crosslinker fraction as a function of elongation; Figure S5: Variation of stress with number of cycles in 100 stretching-unloading cycle tests at 300% strain; Figure S6: Variation of stress with number of cycles in 20 compression-unloading cycle tests at 50% strain; Figure S7: Variation of hysteresis with number of cycles in 20 compression-unloading cycle tests at 50% strain; Figure S8: Nyquist plots of the impedance spectra of the eutectogels over different proportions; Figure S9: Linear sweep voltammetry curve of EG5001 showing the decomposition voltage of around 3.6 V; Table S1: Nomenclature of the ionogels with different polymer mass fractions and monomer molar ratios; Table S2: Summary of the mechanical properties of the ionogels with different compositions at the deformation rate of 100 mm min^−1^ under ambient conditions.
