# Supplementary material for: Highly Transparent, Mechanically Robust, and Conductive Eutectogel Based on Oligoethylene Glycol and Deep Eutectic Solvent for Reliable Human Motions Sensing
Source: Polymers (Basel). 2024 Sep 30;16(19):2761. doi: 10.3390/polym16192761 (PMC11478370; doi:10.3390/polym16192761)
Supplement: Supplementary file 1 [file polymers-16-02761-s001.zip › polymers-3224575-supplementary.pdf]

Supporting Information

for

Highly Transparent, Mechanically Robust, and Conductive Eutectogel Based on Oligoethylene Glycol and Deep Eutectic Solvent for Reliable Human Motions Sensing

Zhenkai Huang[1], Jiahuan Xie[1], Tonggen Li[1], Liguo Xu[4], Peijiang Liu[3,], Jianping Peng[2,*]*

1 School of Materials and Energy, Foshan University, Foshan 528000, China

2 School of Environmental and Chemical Engineering, Foshan University, Foshan 528000, China

3 Reliability Physics and Application Technology of Electronic Component Key Laboratory, the 5th Electronics Research Institute of the Ministry of Industry and Information Technology, Guangzhou 510610, China

4 College of Light Chemical Industry and Materials Engineering, Shunde Polytechnic, Foshan, 528333, China

* Corresponding author

1. Materials and Methods

1.1. Materials

Acrylic acid (AA, 99%) and choline chloride (ChCl, 99%) was purchased from Shanghai Aladdin Scientific Co., Ltd., China. Oligoethylene glycol (OEG) with the average molecular weights of 200 g/mol was obtained from Acros. 2-hydroxy-4'-(2-hydroxyethoxy)-2-methylpropiophenone (Irgacure 2959, 98.0%), and ethylene glycol dimethacrylate (EGDMA, 98.0%) were obtained from Adamas-beta® (Co., Ltd., China). All chemicals were used as received without further purification.

1.2. Preparation of DES based P(AA-ChCl)/OEG eutectogels

The DES based P(AA-ChCl)/OEG eutectogels were prepared by the one-pot photo-initiated free radical polymerization process. To prepare the prepolymer solutions, certain amounts of AA, ChCl, EGDMA, and photo-initiator Irgacure 2959 were dissolved in OEG to form a transparent solution. Different compositions were obtained by varying the mass ratio between different components (see Table S1 for details). The molar percentage of Irgacure 2959 to AA was fixed at 1.0 mol%. The prepolymer solution was put into a vacuum defoaming apparatus for 5 min to remove bubbles, and then transferred into a closed glass mold coated by two polyethylene terephthalate (PET) substrates in the glove box under N_2, and crosslinked under UV light (365 nm) for 1 min.

1.3. Mechanical characterization

Tensile testing was performed on an electronic tensile machine (Instron 5965) with a 100 N load cell in an ambient condition (25 °C, 60% RH), and the stretching rate was set at 100 mm/min. The elastomer samples were cut into a dumbbell shape with a dimension of 20.0 × 2.0 × 1.7 mm^3. The cyclic tensile tests were recorded at a speed of 100 mm/min with a strain of 100%.

1.4. Electrical characterization

The electrical characterization of the eutectogels was performed on an electrochemical workstation (CHI660e, CH Instruments, USA). The initial voltage was the measured open voltage; the frequency ranged from 1 Hz to1 MHz; and the AC amplitude was 0.01 V. The eutectogels were allowed at the desired temperature for at least 30 min to reach the equilibrium before the measurements were taken.

The conductivity (σ) was calculated based on the following equation:

$$\sigma = L / R \times S$$

where L represents the length, R represents the resistance, and S represents the cross-sectional area of the eutectogels.

The strain sensing performance of eutectogels was assessed using an electrochemical workstation in combination with the uniaxial material testing system. Different strains were applied to the eutectogels, and the resulting relative changes in resistance ($\Delta R/R_0$) were obtained, where R_0 represents the resistance of the original eutectogels, R represents the resistance of the stretched eutectogels..

1.5. Fabrication of the flexible strain sensor

The flexible strain sensor was assembled using a rectangle sample with a dimension of 40 × 10 × 1 mm^3. Two ends of the sample were attached with copper foil which connect to the electrochemical workstation, leaving 20 mm ionic conduct distance (*L*) in the middle, and the tensile strain were ranged from 0%-300%. The relative resistance change curves were obtained by the electrochemical workstation (CHI660e, CH Instruments, USA).

1.6. Other characterization

Fourier-transform infrared (FT-IR) spectra were recorded on a PerkinElmer Frontier. Each measurement included an average of about 32 scans from 4000 to 500 cm^{-1}. Optical test was performed on a UV-Vis spectrophotometer (UV-1800 Shimadzu). Ionogel samples with a thickness of 1.0 mm were tested with a wavelength range of 200 to 800 nm. The reference for measuring transparency was air.

Figure S1-S6.

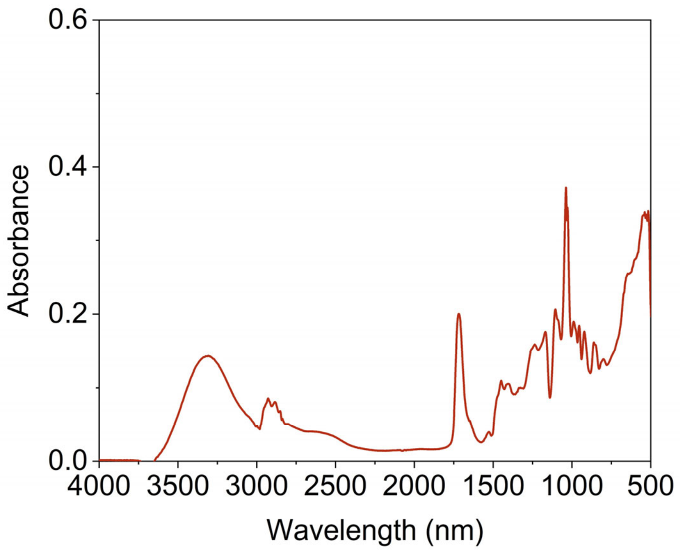

Figure S1. FT-IR spectra of the P(AA-ChCl)/OEG eutectogel with the proportion of EG5001 from wavelength 500 to 4000 cm^{-1}.

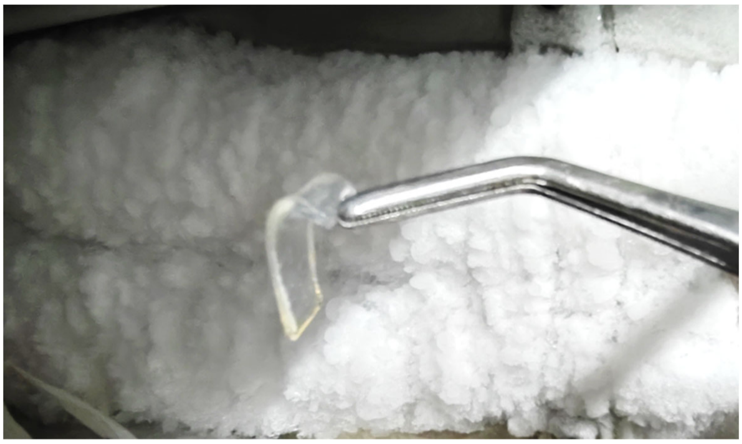

Figure S2. At temperatures of −10 °C, the eutectogel remains its flexibility and bends naturally under gravity.

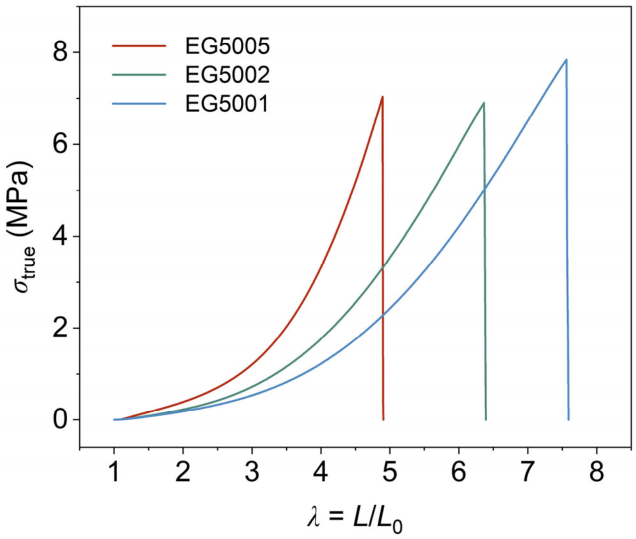

Figure S3. Corresponding true stress-strain curves of the eutectogels with different crosslinker fraction as a function of elongation.

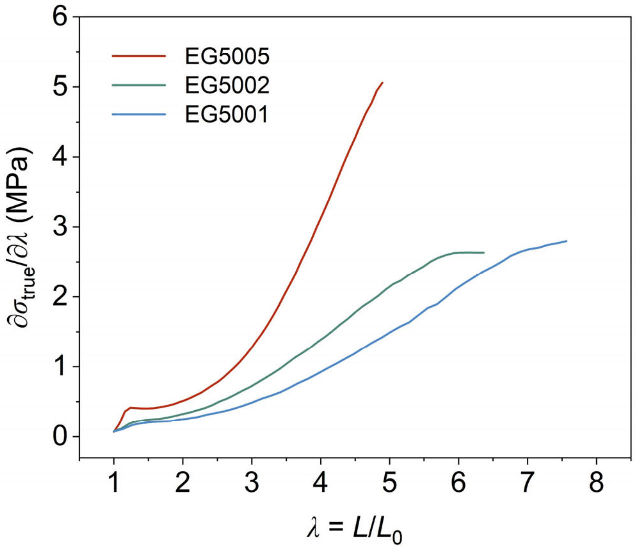

Figure S4. Corresponding differential modulus-strain curves of the eutectogels with different crosslinker fraction as a function of elongation.

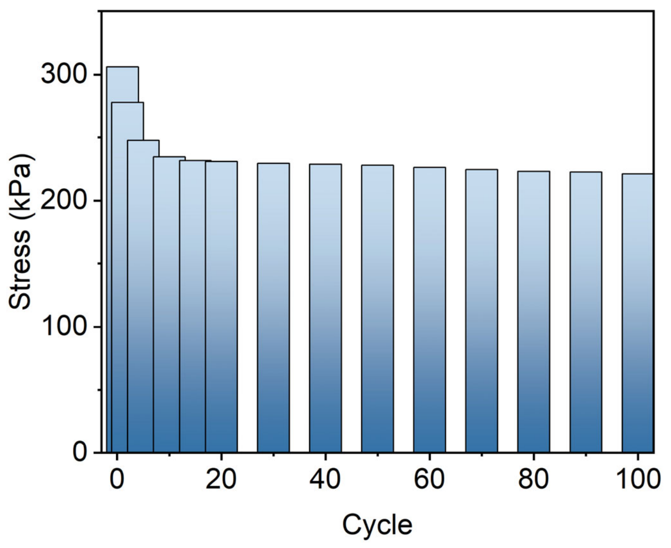

Figure S5. Variation of stress with number of cycles in 100 stretching-unloading cycle tests at 300% strain.

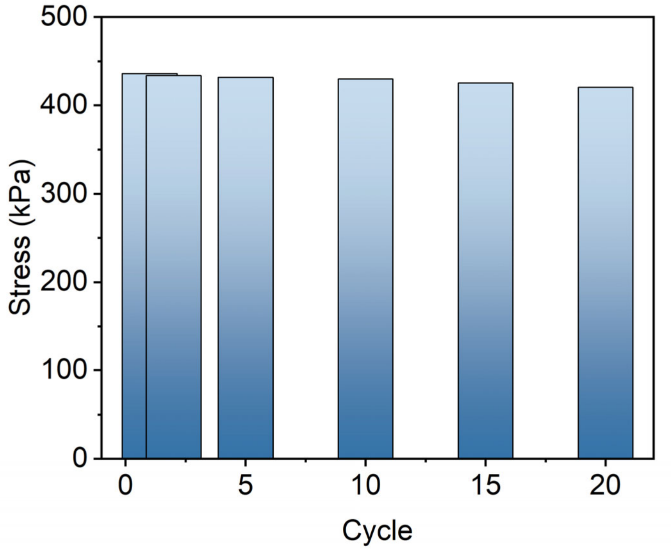

Figure S6. Variation of stress with number of cycles in 20 compression-unloading cycle tests at 50% strain.

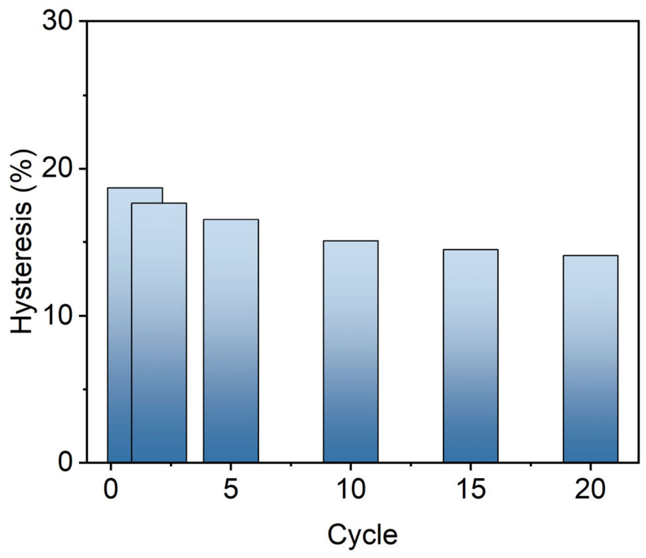

Figure S7. Variation of hysteresis with number of cycles in 20 compression-unloading cycle tests at 50% strain.

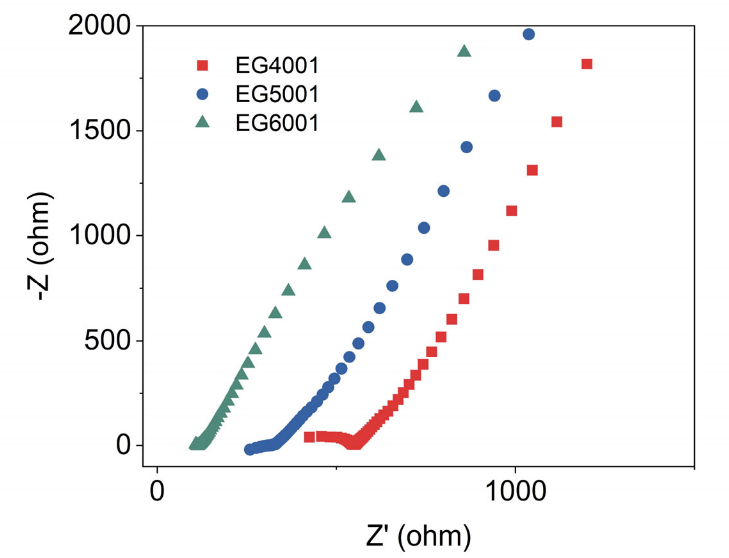

Figure S8. Nyquist plots of the impedance spectra of the eutectogels over different proportions.

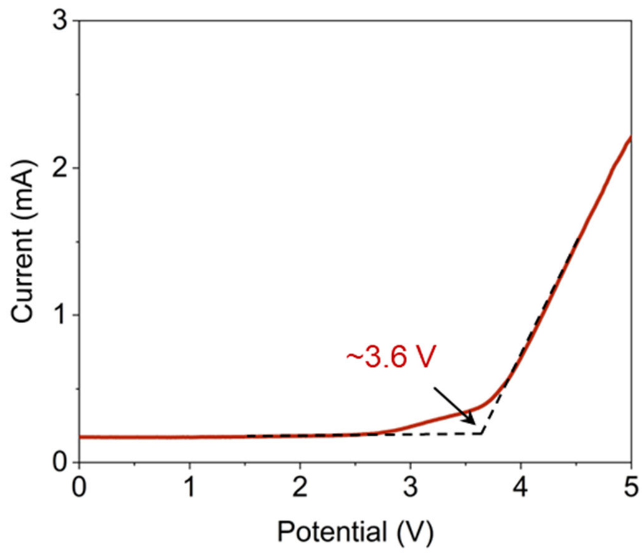

Figure S9. Linear sweep voltammetry curve of EG5001 showing the decomposition voltage of around 3.6 V.

Table S1-S2.

Table S1. Nomenclature of the ionogels with different polymer mass fractions and monomer molar ratios.

Samples	OEG mass fraction (%)	AA to ChCl molar ratio	EGDMA molar ratio (%)
EG3001	30	2:1	0.1
EG4001	40	2:1	0.1
EG5001	50	2:1	0.1
EG5002	50	2:1	0.2
EG5003	50	2:1	0.3
EG6001	60	2:1	0.1
EG7001	70	2:1	0.1

Table S2. Summary of the mechanical properties of the ionogels with different compositions at the deformation rate of 100 mm min^{-1} under ambient conditions.

Composition	Maximum tensile strength (MPa)	Strain at break (%)	Young's modulus (kPa)	Toughness (MJ/m^3)
EG3001	1.52	484	197.5	2.67
EG4001	1.26	545	192.9	2.62
EG5001	1.04	655	55.9	2.79
EG5002	1.09	536	93.9	2.38
EG5003	1.44	389	199.0	1.99
EG6001	0.86	774	24.6	2.18
EG7001	0.66	962	9.5	2.07
